# Physical activity as a treatment for depression: the TREAD randomised trial protocol

**DOI:** 10.1186/1745-6215-11-105

**Published:** 2010-11-12

**Authors:** Helen Baxter, Rachel Winder, Melanie Chalder, Christine Wright, Sofie Sherlock, Anne Haase, Nicola J Wiles, Alan A Montgomery, Adrian H Taylor, Ken R Fox, Debbie A Lawlor, Tim J Peters, Deborah J Sharp, John Campbell, Glyn Lewis

**Affiliations:** 1 School of Social and Community Medicine, University of Bristol, Oakfield House, Oakfield Grove, Bristol, BS8 2BN, UK; 2 Primary Care Research Group, Peninsula Medical School, Smeall Building, St Luke's Campus, Magdalen Road, Exeter, EX1 2LU, UK; 3 School of Policy Studies, University of Bristol, 8 Priory Road, Bristol, BS8 1TZ, UK; 4 School of Sport and Health Sciences, Richards Building, St Luke's Campus, Magdalen Road, Exeter, EX1 2LU, UK; 5 School of Clinical Sciences, University of Bristol, Southmead Hospital, Westbury-upon-Trym, Bristol, BS10 5NB, UK; 6 School of Social and Community Medicine, University of Bristol, Canynge Hall, 39 Whatley Road, Bristol, BS8 2PS, UK

## Abstract

**Background:**

Depression is one of the most common reasons for consulting a General Practitioner (GP) within the UK. Whilst antidepressants have been shown to be clinically effective, many patients and healthcare professionals would like to access other forms of treatment as an alternative or adjunct to drug therapy for depression. A recent systematic review presented some evidence that physical activity could offer one such option, although further investigation is needed to test its effectiveness within the context of the National Health Service.

The aim of this paper is to describe the protocol for a randomised, controlled trial (RCT) designed to evaluate an intervention developed to increase physical activity as a treatment for depression within primary care.

**Methods/design:**

The TREAD study is a pragmatic, multi-centre, two-arm RCT which targets patients presenting with a new episode of depression. Patients were approached if they were aged 18-69, had recently consulted their GP for depression and, where appropriate, had been taking antidepressants for less than one month. Only those patients with a confirmed diagnosis of a depressive episode as assessed by the Clinical Interview Schedule-Revised (CIS-R), a Beck Depression Inventory (BDI) score of at least 14 and informed written consent were included in the study. Eligible patients were individually randomised to one of two treatment groups; usual GP care or usual GP care plus facilitated physical activity. The primary outcome of the trial is clinical symptoms of depression assessed using the BDI four months after randomisation. A number of secondary outcomes are also measured at the 4-, 8- and 12-month follow-up points including quality of life, attitude to and involvement in physical activity and antidepressant use/adherence. Outcomes will be analysed on an intention-to-treat (ITT) basis and will use linear and logistic regression models to compare treatments.

**Discussion:**

The results of the trial will provide information about the effectiveness of physical activity as a treatment for depression. Given the current prevalence of depression and its associated economic burden, it is hoped that TREAD will provide a timely contribution to the evidence on treatment options for patients, clinicians and policy-makers.

Trial registration: ISRCTN 16900744

## Background

Depression is a leading contributor to disability in the UK and is associated with a decrement of health greater than many other chronic diseases [1,2]. It impacts not only upon the life of the individual but also upon their carers, employers and society as a whole [3-5]. Whilst antidepressants are the most commonly prescribed treatment for depression, there is concern about their use and effectiveness, especially in mild/moderate cases [6-8]. Cognitive behavioural therapy and counselling are alternatives or adjuncts to medication, but access to therapists within United Kingdom (UK) primary care is variable and can involve long waiting times. For these reasons, there is a need to identify other effective non-pharmacological interventions for the management of depression.

A recent systematic review presented some evidence that physical activity could be an effective treatment for depression, although much of the research considered had important limitations [9].

Many randomised controlled trials (RCTs) conducted to date have recruited from a non-clinical setting or have offered financial or other incentives to participate [9,10]. Results from such trials are difficult to generalise to patients who present routinely to primary care since community volunteers or paid subjects are likely to display higher levels of motivation than potential participants who are identified within the clinical setting.

Reported trials have frequently been small and of poor quality. Many have used inadequately concealed randomisation procedures or failed to conduct proper intention-to-treat (ITT) analyses, so may have exaggerated any observed treatment effects [9,11]. Studies have also generally been insufficiently powered to detect a meaningful difference between the treatment groups, with even the largest study reporting fewer than 50 participants per treatment arm [12].

Length of follow-up has been problematic, with those studies of shortest duration often reporting the largest effects, suggesting that any impact may be diminished or disappear altogether in the longer term. Indeed, only a small proportion of RCTs have investigated whether any benefits outlasted the duration of the intervention itself [13].

Little is known about the type, intensity or duration of physical activity that might prove effective for the treatment of depression. The context within which any intervention is delivered may also be pivotal in terms of the uptake and sustainability of physical activity for the individual. Furthermore, any intervention will need to consider the feasibility and practicality of delivery, as well as addressing any issues of motivation, energy and self-esteem prevalent within a depressed population.

### Aim

The aim of this paper is to describe the protocol for an RCT designed to evaluate an intervention developed to increase physical activity, as a treatment for depression within primary care.

## Methods/design

### Recruitment of participants and baseline assessment

**Figure 1:**
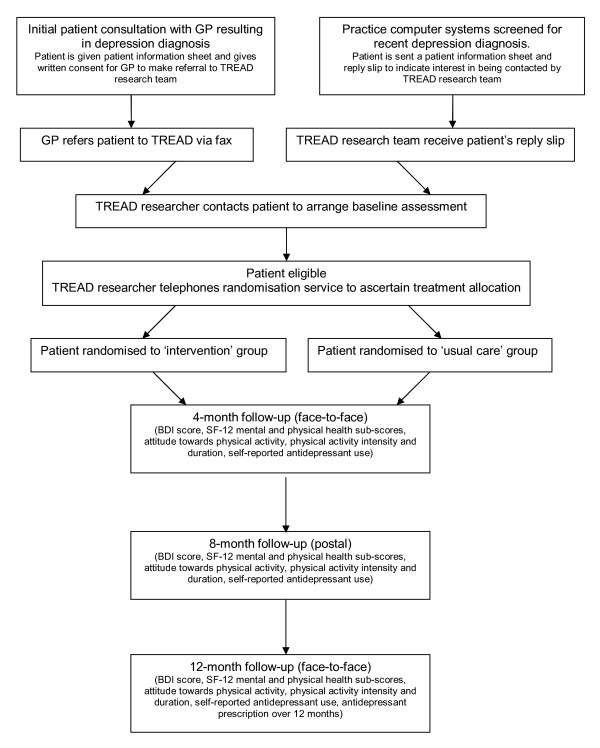
**A flowchart of the TREAD trial design**.

The TREAD study is a pragmatic, multi-centre, two-arm RCT into which patients recently diagnosed with a new episode of depression are recruited from primary care (as shown in Figure 1). Recruitment for the trial took place over a 27-month period between August 2007 and October 2009 in general practices from the Bristol and Exeter areas. Patients could be included if they were aged 18-69, had recently consulted their GP for depression and had either recently started taking antidepressants (within four weeks of their baseline assessment and following an antidepressant free period of at least one month) or were not currently on antidepressants. Ethical approval for the study was given by West Midlands Multi-centre Research Ethics Committee (MREC) and research governance approval was obtained from the relevant local Primary Care Trusts (PCTs).

Individuals who were unable to complete self-administered questionnaires in English, or had medical contraindications to physical activity, psychosis, bipolar disorder or any serious substance abuse problem were excluded from the study. Women pregnant at the time of recruitment were also automatically excluded but those women who became pregnant in the course of their study participation were encouraged to continue under their GP's supervision.

The majority of patients referred to TREAD were identified during routine consultations, when they were given a patient information leaflet by their GP and, if interested, asked to provide written authority to enable further contact by the research team. In some practices, computer systems were also regularly searched for details of patients recently diagnosed as depressed or prescribed an antidepressant, in an effort to alert GPs to potentially eligible individuals. In this instance, patients were sent information about the study from their surgery and encouraged to respond to the research team directly, if interested, using a reply-paid slip. Once a referral was received, a researcher telephoned the patient to introduce the study formally, make initial eligibility checks and to arrange an appointment for baseline assessment. Referred patients were visited at home or at their GP surgery in order to obtain consent and assess eligibility using the Clinical Interview Schedule (CIS-R) [14] and the Beck Depression Inventory (BDI) [15]. Only patients with a confirmed diagnosis of a depressive episode according to the International Statistical Classification of Diseases and Related Health Problems 10th Revision (ICD-10), a BDI score of at least 14 and informed written consent were included in the study.

In addition to the RCT, a nested qualitative study will investigate the experience and acceptability of the intervention for both participants and health professionals. An economic analysis will also be performed using a quality of life measure and information gathered on health service use to assess the cost effectiveness of the intervention.

### Randomisation procedure

Eligible and consenting patients were individually randomised at the end of their baseline assessment to one of two treatment groups; usual GP care or usual GP care plus facilitated physical activity. Randomisation was stratified to take account of antidepressant use (yes, no) and minimised by severity of depression (CIS-R score of ≤25, 26-33, >34 at baseline), recruiting centre (Bristol, Exeter) and level of physical activity (≤1, 2-3, >4 days per week where at least 30 minutes of moderate intensity physical activity was being undertaken). Allocation was concealed from the researcher using an automated telephone randomisation system which was administered remotely and used a computer-generated code.

### Follow-up

Follow-up data collection is scheduled at three time-points; 4, 8 and 12 months post-randomisation. The 4-month follow-up was chosen as the primary outcome time point since it represents the stage in the intervention period at which we expect to observe the largest effect. The 8-month follow-up coincides with the end of the intervention delivery, whilst the 12-month follow-up will enable the investigation of any longer term effects of the intervention on study outcomes. All follow-up data collection employs a self-report research instrument.

In order to maximise retention, researchers meet study participants to supervise the data collection process at the 4 and 12-month follow-up points wherever possible. Due to restricted resources, 8-month follow-up is conducted solely using a postal questionnaire. Any participants unable to attend their 4 and 12-month follow-up sessions are contacted by telephone to arrange a further appointment or sent the questionnaire by post if re-arranging a face-to-face session is difficult.

### Intervention

All study participants are encouraged to follow the advice of their GP regarding depression and its treatment throughout their involvement in the trial. In addition to this, participants in the intervention arm of the study are offered the support of a Physical Activity Facilitator (PAF) over an 8-month period. All PAFs are trained specifically for the study and receive regular supervision and feedback to maximise fidelity to model.

The TREAD intervention is theoretically-driven [16,17] and tailored to address the specific challenges of isolation, apathy and social anxiety which are common within a depressed population. It aims to provide relatively intensive, individually tailored support in a context that will encourage maximum engagement in physical activity. Following a specially developed manual, PAFs use a range of motivational interviewing techniques and goal-setting strategies to encourage participants' uptake of a range of acceptable and locally available physical activities. The rationale and development of the TREAD intervention is discussed in more detail in Haase et al [18].

The intervention comprises an initial hour-long, face-to-face assessment session followed by a series of up to ten short telephone contacts and two further half-hour, face-to-face meetings, with the scheduling left to the discretion of the PAF and the participant. The expectation is that at least five sessions including one face-to-face meeting would be delivered by the primary outcome point i.e. by 4-month follow-up.

### Outcome measures

#### Primary outcome

The Beck Depression Inventory (BDI) [15] is collected by self-report measure at the 4-month follow-up point [15]. The resulting score is treated as both a continuous (range 0 to 63) and binary (where less than 10 indicates recovery) variable in order to provide a quantitative measure of improvement and an estimate of the proportion of patients reaching symptomatic recovery.

#### Secondary outcomes

The longer term effects of the intervention are measured using the BDI at the 8 and 12-month follow-up points and the Short Form-12 Health Survey (SF-12) [19] at 4, 8 and 12-month follow-up points. Attitude to, and involvement in physical activity are also measured by self-report at the same three follow-up points. Antidepressant use is assessed using a self-reported measure of medication adherence at all three follow-up points whilst GPs' records will provide details of antidepressant prescription over the entire 12-month follow-up period.

### Statistical Analysis

The analysis and reporting of this trial will be undertaken in accordance with Consolidated Standards of Reporting Trials (CONSORT) guidelines [20] with the primary comparative analyses conducted on an intention-to-treat (ITT) basis using linear and logistic regression models without imputation of missing outcome data. Descriptive statistics of key clinical and socio-demographic variables will be obtained as a means of detecting any marked imbalance between the randomised groups at baseline, with investigation of the effects on the primary analyses of additional adjustment for any such variables.

The primary outcome measure (BDI score at 4-month follow-up point) will be used and presented in both binary and a continuous form, adjusting for BDI score at baseline and the stratification/minimisation variables. For the continuous outcome, the result will be presented as the (adjusted) difference in mean score between the intervention and control groups. For the binary outcome, the result will be presented as an (adjusted) odds ratio of recovery in the intervention group compared with the control group. Full attention will be paid to 95% confidence intervals as well as p-values.

The BDI score will also be considered in both binary and continuous form in secondary analyses, using data from the 4, 8 and 12-month follow-up points in a repeated measures analyses. These will investigate whether any between-group differences alter over time, and in the absence of any time effect, will yield an average effect over the three follow-up assessments. SF-12 sub-scores, attitude to physical activity, physical activity level and self-reported antidepressant use will be analysed employing appropriate regression techniques at 4-month follow-up and also in a repeated measures analysis conducted using 4, 8 and 12-month follow-up data. Antidepressant prescription data will be analysed as a continuous variable using all data available in an appropriate regression model. Consideration will be given to the adjustment of p-values for multiple testing.

The effect of missing data will be investigated by generating a complete dataset using the Multiple Imputation Chain Equation (MICE) method [21]. Complier-Average Causal Effect (CACE) estimates [22], or treatment efficiency, will be estimated using instrumental variable regression and will compare outcomes for those participants receiving an adequate 'dose' of the intervention with a comparable group of would-be compliers in the control arm. The extent and impact on the results of clustering by general practice will also be investigated.

### Sample size justification

The sample size calculation was based on the BDI score as both a binary and a continuous outcome measure. As it was initially thought that 10% of the population would not be taking antidepressants at the time of recruitment, the intention was to omit these individuals from the primary analysis. The original calculation had estimated that 60% of participants in the usual care group and 73% in the intervention group would have recovered by the 4-month follow-up, that is scoring < 10 on the BDI. A difference of 13% in the proportion 'recovered', equivalent to an odds ratio of 1.8, is consistent with the lower end of treatment effects observed with antidepressant medication and is considered clinically worthwhile. With 90% power and 5% two-sided alpha, 291 recruited patients would be required for each treatment group. Previous studies using the BDI as a continuous outcome have estimated a standard deviation of about 9 points [23] and have suggested a worthwhile and feasible target difference of 3-4 points. Thus, allowing for a maximum attrition rate of 15%, the required sample size was initially calculated to be 762.

However, in the early stages of the trial, the percentage of participants not on antidepressant treatment was found to be almost 50%. Given the implications for total sample size, and that allocation was stratified by antidepressant use, it was proposed that all randomised participants should be included in the primary analysis. In addition, whilst the recovery rate of the participants in the control group was initially assumed to be around 60%, a recently concluded study [24] found that the proportion of participants recovering in the equivalent group was nearer to 20% (95% CI 12.9-30.3). Thus, the original power calculations were revised to reflect a reduced sample size of 360 patients randomised over a 27-month recruitment period.

As shown in Table 1, the revised power calculations provide adequate power for the primary analysis using the continuous outcome and, although there will inevitably be some reduction in power for the binary outcome, the ability to detect a 15% difference with 80% power remains.

**Table 1:** Sample Size Calculation

total N randomised	N for primary analysis	power for 60% vs. 73% (OR = 1.80) ^1^	power for 20% vs. 33% (OR = 1.97) ^2^	detectable difference with 80% power ^3^	power to detect 3 BDI point difference
360	306	63%	69%	15%	82%

^1 ^based on original sample size

^2 ^revised calculation based on data from the IPCRESS study [24]

^3 ^percentage difference of intervention group from usual care group assuming 20% recovery in usual care group

## Discussion

This study has been designed to address the limitations highlighted in previous research on the subject of physical activity as a treatment for depression. All participants are recruited directly from within the primary care setting, thereby avoiding any undue influence from incentivisation and allowing the results to inform clinical practice. The study uses a remote randomisation system to protect concealment of allocation and proposes that the primary comparative analyses be conducted on an ITT basis in accordance with the CONSORT guidelines [20].

A two-arm design, with 180 participants per group should provide sufficient power to detect a meaningful difference in outcome. There will also be important supplementary CACE analyses to estimate efficiency in the presence of non-compliance with the intervention. All participants will be followed-up for a 12-month period, in order to consider the impact of the intervention in both the immediate and longer term. Finally, the intervention has been specifically developed to extend the scope of existing successful interventions and has robust theoretical underpinnings.

Physical activity, if found to be effective, could be an important alternative or adjunct treatment for depression, particularly for those who prefer non-pharmacological interventions. Currently, however, little is known about the mechanisms that might mediate any therapeutic effects of physical activity on depression. There are a number of hypothesised biological and psychosocial mechanisms but it is likely that an effective physical activity intervention would rely on multiple mechanisms. TREAD aims to evaluate, in general terms, whether physical activity can be an effective treatment for depression within primary care. Future research might identify which particular mechanisms, and any interactions between them, are most effective, as well as determining the optimum type, intensity and duration of physical activity required to produce a therapeutic effect.

### Current Study Status

The TREAD trial began recruiting patients in August 2007 and closed to recruitment in October 2009. Data collection is due to be completed in November 2010 and results will be published in February 2011.

## List of Abbreviations

BDI: Beck Depression Inventory; CACE: Complier-Average Causal Effect; CIS-R: Clinical Interview Schedule-Revised; CONSORT: Consolidated Standards of Reporting Trials; DH: Department of Health; DMC: Data Monitoring Committee; GP: General Practitioner; HTA: Health Technology Assessment; ICD-10: International Statistical Classification of Diseases and Related Health Problems 10th Revision; ITT: Intention-to-treat; MHRN: Mental Health Research Network; MICE: Multiple Imputation Chain Equation; MREC: Multi-centre Research Ethics Committee; NHS: National Health Service; NICE: Nation Institute of Clinical Excellence; NIHR: National Institute for Health Research; PCT; Primary Care Trusts; RCT: Randomised Controlled Trial; SF-12: 12-item Short Form Health Survey; TSC: Trial Steering Committee; UK: United Kingdom;

## Competing interests

DS and JC are currently working as General Practitioners whilst GL is a Psychiatrist. All have endeavoured to ensure that their research has not been biased by their own clinical practice.

## Authors' contributions

AH, NW, AM, AT, KF, DL, TP, DS, JC and GL were responsible for the initial protocol, securing funding for the trial and refinement of the protocol. MC is the trial co-ordinator responsible for the ongoing management of the trial and contributed to the refinement of the protocol. MC is also using data from the study for her PhD research, MC and HB conducted the feasibility phase work and prepared all trial materials. HB, RW, CW, SS were responsible for the recruitment of patients and general practices to the study and the majority of the follow-up data collection. Analysis and interpretation of data is being conducted by MC with the assistance of NW, AM, TP and GL. HB and RW wrote the initial draft of the manuscript. All authors contributed to and approved the final manuscript on behalf of the wider research team.

## References

[B1] Moussavi S Chatterji S Verdes E Tandon A Patel V Üstun B Depression, chronic diseases and decrements in health: results from the World Health Surveys The Lancet 2007 370 808 9 10.1016/S0140-6736(07)61415-9 17826170

[B2] McCormick A Fleming D Charlton J Morbidity Statistics from General Practice Fourth National Study 1991-1992

[B3] Üstun TB Ayuso-Mateos JL Chatterji S Mathers C Murray CJL Global burden of depressive disorders in the year 2000 British Journal of Psychiatry 2004 184 386 392 10.1192/bjp.184.5.386 15123501

[B4] Singleton N Bumpsted R O'Brien M Lee A Meltzer H Psychiatric morbidity among adults living in private households London: HMSO 2001 10.1080/0954026021000045967 12745312

[B5] Lépine J Gastpar J Mendlewicz J Tylee A on behalf of the DEPRES Steering Committee Depression in the community: the first pan-European study DEPRES (Depression Research in European Society) International Clinical Psychopharmacology 1997 12 19 29 10.1097/00004850-199701000-00003 9179630

[B6] National Institute for Clinical Excellence Depression: management of depression in primary and secondary care Clinical Guideline 23 http://www.nice.org.uk/nicemedia/live/12329/45888/45888.pdf (accessed 27th August 2010)

[B7] Pigott HE Leventhal AM Alter GS Boren JJ Efficacy and effectiveness of antidepressants: current status of research Psychotherapy and Psychosomatics 2010 79 267 79 10.1159/000318293 20616621

[B8] Hermens MLM van Hout HPJ Terluin B Ader HJ Penninx BWJH van Marwijk HWJ Bosmans JE van Dyck R de Hann M Clinical effectiveness of usual care with or without antidepressant medication for primary care patients with minor or mild-major depression: a randomized equivalence trial BMC Medicine 2007 5 5 36 10.1186/1741-7015-5-36 18067659 PMC2234409

[B9] Mead GE Morley W Campbell P Greig CA McMurdo M Lawlor DA Exercise for depression The Cochrane Database of Systematic Reviews 2009 3 Art No.: CD004366 10.1002/14651858.CD004366.pub4 19588354

[B10] Lawlor D Hopker S The effectiveness of exercise as an intervention in the management of depression: systematic review and meta-regression analysis of randomized controlled trials British Medical Journal 2001 322 763 67 10.1136/bmj.322.7289.763 11282860 PMC30551

[B11] Schulz KF Chalmers I Hayes RJ Altman DG Empirical evidence of bias: dimensions of methodological quality associated with estimates of treatment effects in controlled trials Journal of American Medical Association 1995 273 408 12 10.1001/jama.273.5.408 7823387

[B12] Blumenthal JA Babyak MA Doraiswamy PM Watkins L Hoffman BM Barbour KA Herman S Craidhead E Brosse AL Waugh R Hinderliter A Sherwood A Exercise and Pharmacotherapy in the treatment of major depressive disorder Psychosomatic Medicine 2007 69 587 96 10.1097/PSY.0b013e318148c19a 17846259 PMC2702700

[B13] Krogh J Nordentoft M Sterne JAC Lawlor D The effect ofexercise on clinically depressed adults: Systematic review and meta-analysis of randomized controlled trials Journal of Clinical Psychology 10.4088/JCP.08r04913blu 21034688

[B14] Lewis G Assessing psychiatric disorder with a human interviewer or computer Journal of Epidemiology and Community Health 1994 48 207 10 10.1136/jech.48.2.207 8189180 PMC1059935

[B15] Beck AT Ward CH Mendelsohn M Mock J Erbaugh J An inventory for measuring depression Archives of General Psychiatry 1961 4 561 71 13688369 10.1001/archpsyc.1961.01710120031004

[B16] Deci EL Ryan RM Intrinsic motivation and self-determination in human behaviour 1985 New York: Plenum Press

[B17] Glaser B Strauss A The discovery of grounded theory 1967 Chicago: Aldine

[B18] Haase A Taylor AH Fox KR Thorp H Lewis G Rationale and development of the physical activity counselling intervention for a pragmatic trial of exercise and depression in the UK Mental Health and Physical Activity 2010

[B19] Moher D Schulz KF Altman DG The CONSORT statement: Revised recommendations for improving the quality of Reports of Parallel-Group randomized trials The Lancet 2001 357 1191 4 10.1016/S0140-6736(00)04337-3 11323066

[B20] Ware JE Sherbourne CD The MOS 36-item short-form health survey (SF-36): I. Conceptual framework and item selection Medical Care 1992 30 483 1593914

[B21] van Buuren S Boshuizen HC Knock DL Multiple imputation of missing blood pressure covariates in survival analysis Statistical Medicine 1999 18 681 94 10.1002/(SICI)1097-0258(19990330)18:6<681::AID-SIM71>3.0.CO;2-R 10204197

[B22] Dunn G Maracy M Tomenson B Estimating treatment effects from randomized clinical trials with non-compliance and loss to follow-up: the role of instrumental variable methods Statistical Methods Medical Research 2005 14 369 96 10.1191/0962280205sm403oa 16178138

[B23] Elkin I Shea T Watkins JT Imber SD Sotsky SM Collins JF Glass DR Pilkonis PA Leber WR Docherty JP Fiester SJ Parloff MB National Institute of Mental Health Treatment of Depression Collaborative Research Program: general effectiveness of treatments Archives of General Psychiatry 1989 46 971 83 2684085 10.1001/archpsyc.1989.01810110013002

[B24] Kessler D Lewis G Kaur S Wiles N King M Weich S Sharp D Araya R Hollinghurst S Peters TJ Therapist-delivered internet psychotherapy for depression in primary care: a randomised controlled trial The Lancet 374 628 634 10.1016/S0140-6736(09)61257-5 19700005

